# Comprehensive Analysis of the GXXXG Motif Reveals Structural Context-Dependent Diversity and Composition Across Proteins

**DOI:** 10.3390/ijms26189014

**Published:** 2025-09-16

**Authors:** Chi-Jen Lo, Ting-Fong Lin, Yue-Li Juang, Yi-Cheng Chen

**Affiliations:** 1 Metabolomics Core Laboratory, Heathy Aging Research Center, Chang Chung University, Taoyuan 333, Taiwan; chijenlo@gmail.com; 2 Institute of Biomedical Sciences, MacKay Medical University, New Taipei City 250, Taiwan; tan03654@hotmail.com (T.-F.L.); yljylk@mmu.edu.tw (Y.-L.J.); 3 School of Medicine, College of Medicine, MacKay Medical University, New Taipei City 250, Taiwan

**Keywords:** GXXXG, glycine-zipper motif, TM, non-TM, shared motif

## Abstract

The GXXXG motif, also called the glycine zipper, is a common sequence pattern that facilitates tight packing of secondary structures, especially through helix–helix interactions in both membrane and soluble proteins. However, its overall distribution, sequence variation, and structural preferences depending on context are not fully understood. Here, we offer a detailed, large-scale analysis of GXXXG motifs, examining over 25,000 unique UniProt sequences with structural data. We classified the motifs as transmembrane (TM), non-transmembrane (non-TM), or shared, based on their TM coverage, and analyzed them via statistical models, diversity measures, and compositional profiling. Our findings show that ≥60% TM coverage is a reliable cutoff to distinguish TM-specific motifs, which tend to have less sequence diversity, lower entropy, more hydrophobic residues (notably leucine, isoleucine, and valine), and rank–frequency distributions that follow a heavy-tailed pattern, indicating strong selective pressure. Conversely, non-TM motifs are more varied, with higher entropy and a preference for polar or flexible residues. Shared motifs have intermediate features, reflecting their functional versatility. Power-law and Zipfian analyses support the distinct statistical signatures of TM and non-TM motifs at the 60% coverage threshold. These results enhance our understanding of the structural and evolutionary roles of the GXXXG motif, setting clear standards for identifying TM-specific motifs and offering insights into membrane protein biology, synthetic design, and functional annotation.

## 1. Introduction

Proteins are vital macromolecules involved in numerous biological roles, such as catalyzing reactions, transmitting signals, transporting substances, and providing structural integrity. Their impressive functional range stems from diverse amino acid sequences, folding patterns, and specific sequence motifs—short, recurring patterns that confer unique structural or functional traits. The GXXXG motif, also called the glycine zipper, is especially notable for facilitating tight packing of secondary structures, especially α-helices [1].

The GXXXG motif consists of two glycine residues separated by three amino acids (X), forming a five-residue sequence that promotes inter-helical interactions [1,2]. Its significance in structure arises from glycine’s unique properties, being the smallest amino acid with a side chain of only a hydrogen atom. This minimal bulk allows neighboring helices to come closer together than usual, enabling tight packing via van der Waals forces and hydrogen bonds. This zippering mechanism stabilizes dimeric or oligomeric helix assemblies, playing a key role in maintaining protein structure and function [3].

Initially identified in transmembrane (TM) proteins, the GXXXG motif is crucial for forming and stabilizing membrane-spanning α-helices [4,5,6,7]. For instance, in glycophorin A, this motif facilitates helix–helix dimerization within the membrane’s hydrophobic core, creating stable complexes vital for membrane organization and signaling [7]. Mutational analyses in glycophorin and other TM proteins reveal that replacing either glycine with bulkier amino acids disrupts packing and weakens helix association, highlighting the motif’s functional importance. These studies confirm the GXXXG motif as a key feature of TM helix interactions [8,9,10,11].

Subsequent analyses have shown that GXXXG motifs are not limited to TM proteins. They are also found in soluble proteins and non-membrane regions, where their roles are less clearly understood but probably involve flexibility, disorder, or temporary interactions [2,12,13]. Glycine-rich motifs contribute to conformational flexibility and support dynamic interactions between proteins or between proteins and nucleic acids, demonstrating their versatility beyond membrane contexts [14,15,16]. This finding prompts important questions about how the sequence environment and structural setting affect the presence and variety of GXXXG motifs.

From an evolutionary standpoint, the GXXXG motif demonstrates the balance between functional constraints and sequence diversity. The preservation of glycine at positions one and five indicates a selective pressure to preserve helix packing, while the three X positions allow various residues. However, the amino acid identity at X1, X2, and X3 is not arbitrary: in TM proteins, hydrophobic residues at these spots enhance compatibility with the lipid bilayer and facilitate helix association [1,2,4]. Conversely, soluble proteins tend to have more polar and flexible residues at these positions. These context-dependent preferences imply that the structural and functional roles of the protein shape the allowable sequence variations of GXXXG motifs [3].

Despite decades of research, a comprehensive understanding of how GXXXG motifs are distributed across different proteins, along with how their sequence composition and diversity vary by structural context, remains elusive. Past studies have mostly concentrated on individual proteins or families, emphasizing the motif’s role in specific biological systems, such as in amyloidogenic peptides, where GXXXG motifs facilitate pathological aggregation [17,18]. Other research has documented glycine zippers in TM proteins, but few have thoroughly compared their occurrence, diversity, and sequence preferences between TM, non-TM, and shared environments [4,19].

Extensive protein sequence and structure databases, such as UniProt [20] and the Protein Data Bank [21], now enable systematic research. These resources supply high-quality sequences, structural data, and annotations, including TM topology predictions and experimental structural details. By integrating computational tools for motif detection, redundancy reduction, and statistical analysis, researchers have a valuable opportunity to investigate the overall features of the GXXXG motif.

While the GXXXG motif is well-known for mediating helix–helix interactions in transmembrane proteins, its structural role in soluble contexts remains less defined. This study does not attempt to assign structural or functional roles to individual motifs but rather focuses on their statistical distribution across transmembrane status and taxonomic classifications.

The main goals in this study were to examine how the GXXXG motif is distributed among TM, non-TM, and shared regions, and how its diversity evolves with increased TM coverage. We also explored sequence preferences at positions X1, X2, and X3 in different contexts and what these reveal about functional constraints. Additionally, we investigated whether there is a TM coverage threshold that reliably distinguishes TM-specific motifs.

To answer these questions, we performed a large-scale, quantitative analysis of the GXXXG motif across various structural environments. Our approach included frequency and diversity analysis, Zipf’s law and power-law modeling, information–theoretic divergence and entropy measures, and detailed compositional profiling. The results offer a comprehensive view of the GXXXG motif landscape, emphasizing its evolutionary flexibility and functional adaptation in different structural contexts. We identified TM ≥ 60% as a key threshold for TM-specific motifs and observed distinct preferences for shared motifs. This research enhances our understanding of how sequence–structure relationships influence protein function.

## 2. Results

### 2.1. Dataset Preparation and Processing

The resulting dataset, without further redundancy, contains the following columns: UniProt ID—an identifier from UniProt; PDB ID—an identifier of the protein structure from PDB; Chain ID—a specific chain within the PDB entry; Sequence—an amino acid sequence of the protein containing the GXXXG motif. The final PDF file (Supplementary Data) contains 25,615 UniProt IDs with at least one GXXXG motif. It provides an annotated dataset suitable for further bioinformatic analyses, including structural studies, comparative analyses, and motif conservation assessments. This automated workflow successfully extracts and annotates sequences featuring the GXXXG motif from UniProt, aligns them with structural data from PDB, and facilitates future structural and functional investigations.

The original dataset was enhanced by adding annotations from a taxonomy-rich redundant dataset, which offered considerable biological context, significantly improving interpretability and subsequent analyses. Additional redundancy management, focused on the identical sequence, organism, and UniProt ID, successfully streamlined the dataset by clearly marking redundant entries.

The refined dataset significantly reduces redundancy by thoroughly filtering minor mutation variants using a robust MSA-based method. It includes clear visualization and statistical tracking of the redundancy removal process. The resulting dataset contains 25,434 sequences featuring the GXXXG motif. The final redundant dataset was used to extract the single GXXXG sequence directly from the overall sequence. All other information, including the UniProt ID, PDB ID, Chain ID, organism, taxonomy, and the start position of the GXXXG sequence, is also included.

### 2.2. Statistical and Summary of GXXXG Motif

**Figure 1:**
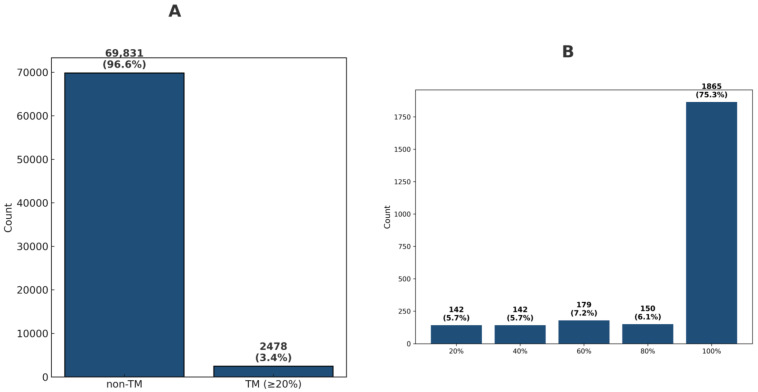
The distribution of GXXXG motifs in TM and non-TM regions. (**A**) The number of GXXXG motifs categorized as non-transmembrane (non-TM) and transmembrane (TM, with ≥20% coverage). (**B**) The proportion of TM motifs at various TM coverage thresholds (20%, 40%, 60%, 80%, and 100%). The group with 100% TM coverage is the most common among TM motifs, making up about 75% of TM-annotated motifs.

**Figure 2:**
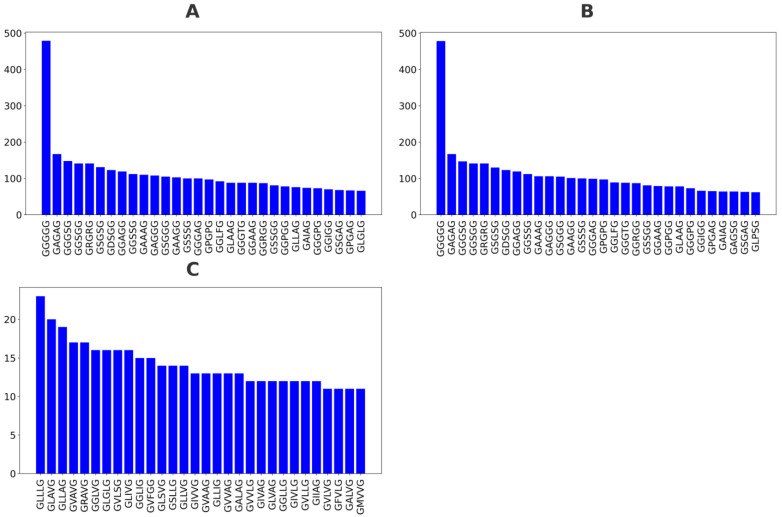
The 30 most frequent GXXXG motifs in various structural contexts. (**A**) The top 30 GXXXG motifs overall across all sequences. (**B**) The top 30 motifs found only in non-TM regions. (**C**) The top 30 motifs in TM regions with at least 20% coverage. Motifs are ordered by frequency; hydrophobic-rich motifs are more prevalent in TM regions, while glycine-rich motifs are dominant in non-TM regions.

A total of 72,309 GXXXG motifs were obtained. Using the UniProt XML transmembrane annotation, the GXXXG motif dataset was further employed to determine whether they are located in the TM region. A total of 69,831 and 2478 GXXXG motifs were characterized into the non-TM and TM (with TM coverage ≥ 20%) annotations (Figure 1A), respectively. The top 30 GXXXG sequences by frequency for total, non-TM, and TM categories are depicted in Figure 2A, Figure 2B and Figure 2C, respectively.

Further analysis categorized the GXXXG motif into TM regions with coverage of 20%, 40%, 60%, 80%, and 100%. Figure 1B illustrates the distribution of TM coverage percentages. The 100% TM coverage is predominant in the TM category, with a distribution of 75.3%. The distribution percentages for 80%, 60%, 40%, and 20% coverages are 6.1%, 7.2%, 5.7%, and 5.7%, respectively.

To investigate the compositional shift of GXXXG motifs between transmembrane (TM) and non-transmembrane (non-TM) regions, we compared the top 30 most frequent GXXXG motifs from three datasets: all GXXXG motifs combined (Figure 2A), those found only in non-TM regions (Figure 2B), and those found in TM regions with coverage ≥ 20% (Figure 2C).

In the global dataset, the most dominant motif was GGGGG, followed by GAGAG, GGGSG, and GRGRG. The sequence GGGGG appears most frequently, with 479 occurrences, and a total of sixteen sequences have over 100 occurrences. These motifs are highly enriched in glycine and small residues, consistent with the flexible or disordered tendencies of GXXXG regions. Notably, glycine-rich combinations, such as GGGGG, GAGAG, GGGSG, and GSSSG, account for a significant portion of the top entries. This dominance suggests that in the overall dataset, motifs with conformational flexibility are favored. The non-TM-specific motif distribution closely mirrors that of the global dataset. GGGGG remains the most abundant motif, and motifs such as GAGAG, GGGSG, GSSSG, and GRGRG still hold dominant ranks. The overall amino acid composition continues to favor small, polar, or flexible residues, like glycine, serine, and alanine. Importantly, this similarity between the total and non-TM datasets implies that non-TM motifs contribute heavily to the global pattern, potentially overwhelming TM-specific signals.

In contrast, the distribution for motifs from TM regions with ≥20% TM coverage shifts dramatically. While GLLLG still appears and some glycine-rich motifs remain, the dominant motifs are now more hydrophobic and less glycine-rich. The top motifs include GLLLG, GLAVG, GLLAG, GRAVG, and GVAVG, featuring increased occurrences of leucine (L), valine (V), isoleucine (I), and alanine (A)—all hydrophobic residues preferred in membrane-spanning helices. Conversely, the GGGGG sequence was not found in the top 30 GXXXG motifs of the TM category, suggesting that the GGGGG motif is not the most favorable in a transmembrane context. In the TM category, the distribution of GXXXG motifs is more balanced, lacking any motif that far exceeds the others, such as GGGGG.

### 2.3. Unique GXXXG Motif Analysis

**Figure 3:**
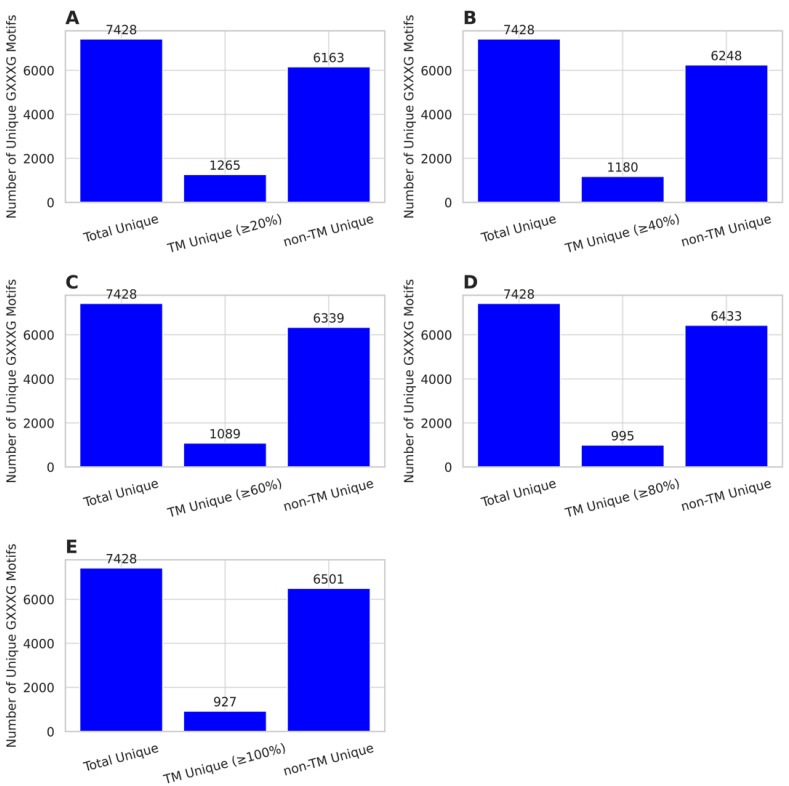
Unique GXXXG motif diversity across TM coverage thresholds. (**A**–**E**) Number of unique GXXXG motifs in TM regions at thresholds of ≥20% (**A**), ≥40% (**B**), ≥60% (**C**), ≥80% (**D**), and ≥100% (**E**), compared to non-TM regions. The diversity of TM motifs decreases as TM coverage increases, whereas non-TM motif diversity stays constant.

To further investigate the diversity of GXXXG motifs, we quantified and compared the number of unique motifs observed in total, TM (≥20% to 100%), and non-TM regions (Figure 3A–E), and TM (20% only to 100% only) and non-TM. Across all coverage thresholds, the total dataset contained 7428 unique GXXXG motifs. However, the number of unique motifs in TM regions decreases markedly as TM coverage stringency increases.

At the TM ≥ 20% level, we identified 1265 unique motifs within TM regions, compared to 6248 unique motifs in non-TM sequences (Figure 3A). Increasing the TM threshold to ≥40% reduced the TM-unique motif count to 1180, a drop of 6.7% (Figure 3B). Further raising the threshold to ≥60% decreased the unique count to 1089 (Figure 3C), while TM ≥ 80% yielded only 995 unique motifs (Figure 3D). At the strictest cutoff (TM ≥ 100%), only 927 motifs remained TM-specific (Figure 3E).

These trends highlight a consistent decline in motif diversity within TM regions as the required TM coverage increases, suggesting that higher TM content selects for a narrower range of sequence motifs. In contrast, the number of unique non-TM motifs remains relatively stable (6163–6501), indicating that motif variability in soluble regions is much broader and less constrained. Interestingly, although the total motif space remains constant (7428 motifs), the TM-specific subset becomes increasingly selective with higher coverage. This implies stronger sequence constraints for full-length membrane-spanning regions, possibly due to structural or biophysical limitations on allowable residue combinations in the membrane environment.

### 2.4. Zipf Analysis of GXXXG Motif Distribution in TM vs. Non-TM Contexts Across Coverage Thresholds

To investigate the statistical characteristics distinguishing TM and non-TM GXXXG motifs, we applied a series of distributional analyses across multiple TM coverage thresholds (20%, 40%, 60%, 80%, and 100%). These analyses included Zipf’s law fitting, entropy evaluation, and information–theoretic divergence measures, such as KL divergence and JSD. For quantitative tail inference, we estimated x_min_ and fit Zipf only on the high-count tail (x ≥ x_min_), corresponding to the low ranks in the rank–frequency view.

**Figure 4:**
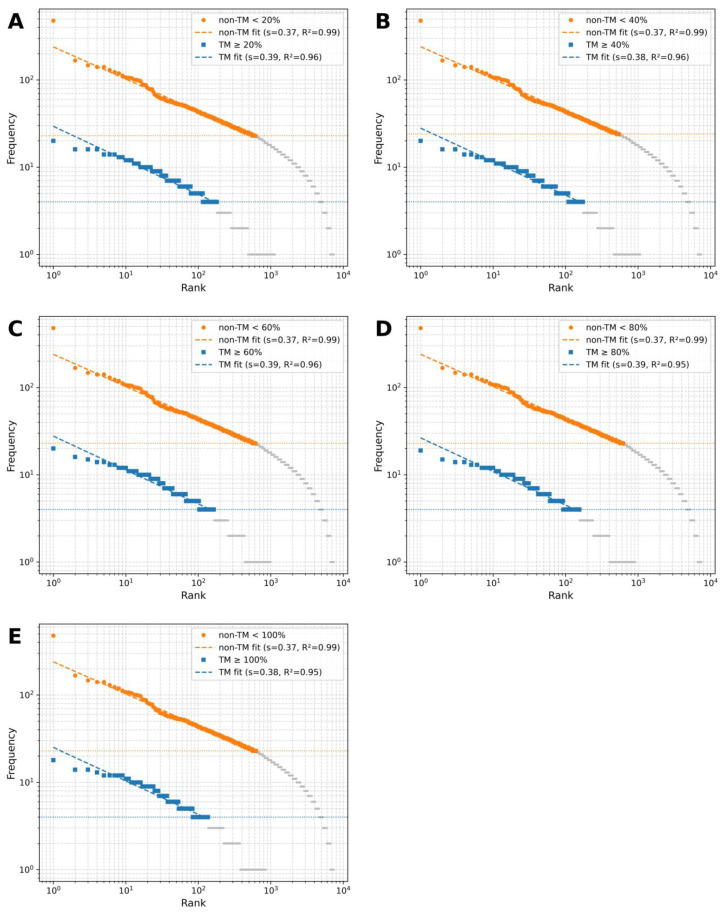
Zipf rank–frequency plots of GXXXG motifs. Log–log rank–frequency graphs for TM and non-TM motifs at coverage thresholds of ≥20% (**A**), ≥40% (**B**), ≥60% (**C**), ≥80% (**D**), and ≥100% (**E**).

**Figure 5:**
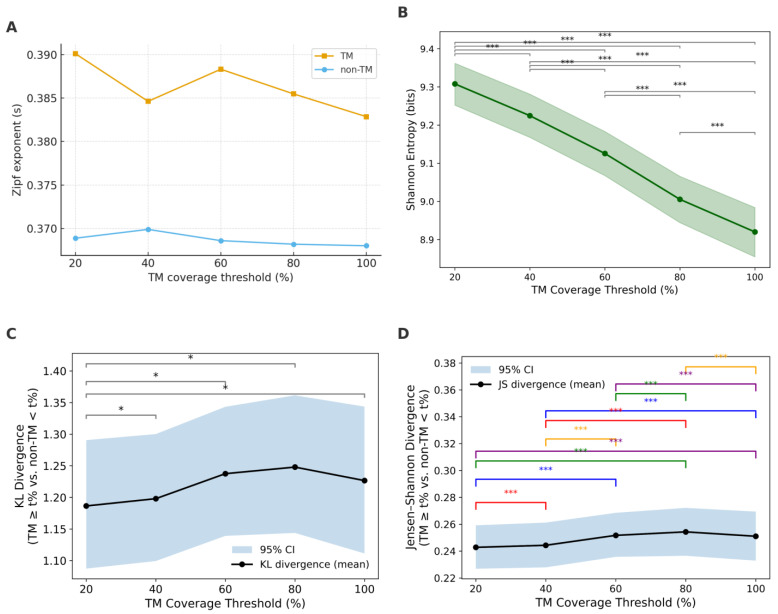
Summary of Zipf, entropy, and divergence metrics across TM coverage thresholds. (**A**) Zipf exponent for TM and non-TM motifs. (**B**) Shannon entropy values of motif distribution with 95% confidence (green shading area) in each category. (**C**) Kullback–Leibler divergence and (**D**) Jensen–Shannon divergence between TM percentages and non-TM distributions with 95% confidence (blue shading area). (* *p* < 0.05, *** *p* < 0.001).

Figure 4A–E show log–log rank–frequency (Zipf) plots for TM coverage thresholds of ≥20%, ≥40%, ≥60%, ≥80%, and ≥100%. Zipf fits were restricted to the high-count region x ≥ x_min_ (horizontal dotted lines), where x_min_ was taken from the power-law analysis (see Section 2.5). Both TM and non-TM motif distributions display heavy-tailed behavior. However, the Zipf exponents |s| distinguish the two groups across all thresholds: non-TM is consistently around s ≈ 0.37 (R^2^ ≈ 0.99), whereas TM is slightly larger, s ≈ 0.38–0.39 (R^2^ ≈ 0.95–0.96) (Figure 5A). This means the TM curves decay a bit more steeply with rank, indicating a slightly more concentrated set of top motifs, while non-TM is marginally flatter (more even) across ranks. Consistently, the power-law tail exponents are lower for TM (α ≈ 3.06–3.19) than for non-TM (α ≈ 3.77–3.79), matching the expected inverse relationship between α and s.

Entropy, as a measure of sequence diversity, further supports the Zipf analysis. The entropy of non-TM motifs remains consistently high (~12.2), reflecting high sequence heterogeneity. In contrast, TM motifs display substantially lower entropy, indicating restricted sequence diversity possibly driven by structural constraints of the membrane environment. Shannon entropy decreased monotonically with increasing TM coverage (Figure 5B), from ~9.7 at TM ≥ 20% to ~9.3 at TM = 100%. All pairwise comparisons were highly significant (*p* < 0.001), indicating progressively reduced sequence diversity at higher TM thresholds. The steepest decline occurred between 40% and 60%, suggesting that motifs beyond 60% TM coverage become notably more homogeneous.

Divergence analyses further supported this transition. KL divergence (TM vs. non-TM) peaked at 40% (~1.28) and declined at higher thresholds (Figure 5C). This indicates that the greatest statistical dissimilarity between TM and non-TM motifs occurs at intermediate thresholds. Jensen–Shannon divergence (Figure 5D) followed a similar non-monotonic pattern: it rose slightly from ~0.25 at 20% to ~0.26 at 40%, remained stable around 60%, and then decreased at higher thresholds (80–100%). Thus, the strongest TM–non-TM separation is observed at intermediate TM coverage rather than at the extremes.

As shown in the Zipf plots, highly frequent motifs like GGGGG and GAGAG are prevalent in the non-TM distribution but are mostly missing in datasets with high TM coverage. Meanwhile, motifs such as GLLLG and GALAG become more prominent only when TM coverage surpasses 60%. This qualitative observation supports the divergence analyses, indicating the development of functionally distinct motif families at higher TM coverage.

Together, these results indicate that TM motifs not only lose entropy with increasing coverage but also diverge systematically from non-TM motifs, with the most pronounced transition occurring between 40% and 60% coverage.

### 2.5. Power Law Analysis of GXXXG Motif Distribution in TM vs. Non-TM Contexts Across Coverage Thresholds

To complement our Zipf analysis of GXXXG motif distributions, we used power-law modeling to examine the statistical behavior of motif frequencies across non-TM and coverage thresholds of ≥20%, ≥40%, ≥60%, ≥80%, and ≥100%. This method provides a more formal way to determine whether these distributions are scale-free and whether the heavy tails in motif counts follow a power-law or other models. Furthermore, power-law modeling offers parameter estimates that describe these distributions, providing an additional perspective alongside Zipf analysis.

**Figure 6:**
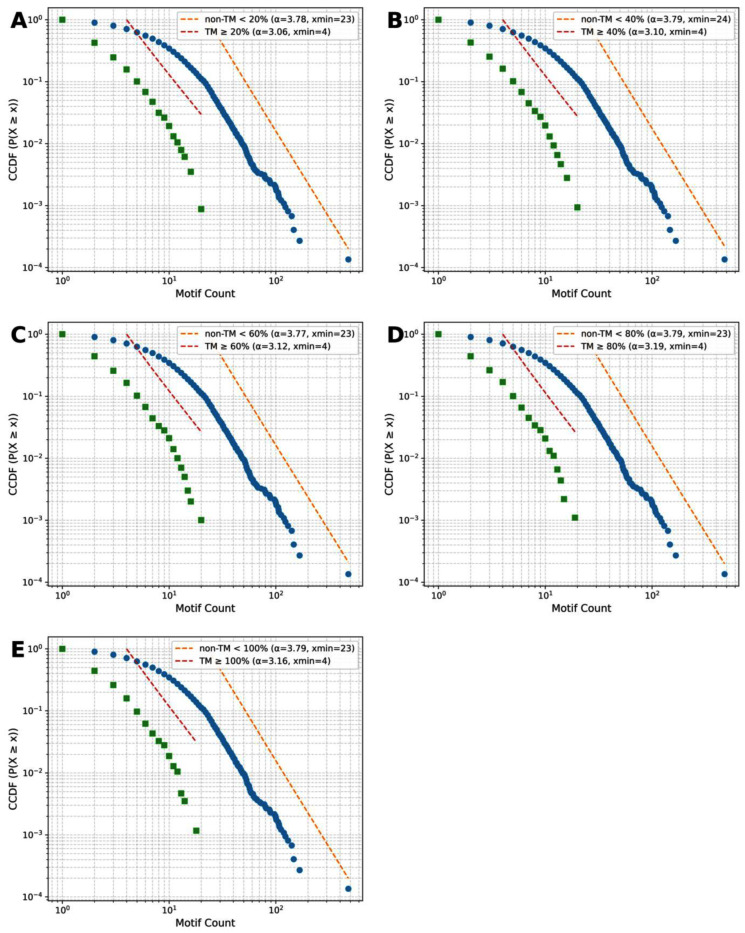
Power-law distributions of GXXXG motif frequencies. Complementary cumulative distribution functions (CCDFs) and power-law fits for TM (green dots) and non-TM (blue dots) motifs at TM coverage thresholds of ≥20% (**A**), ≥40% (**B**), ≥60% (**C**), ≥80% (**D**), and ≥100% (**E**).

Figure 6A–E illustrate the complementary cumulative distribution functions (CCDFs) of motif counts on log–log axes, across TM coverage thresholds of ≥20%, 40%, 60%, 80%, and 100%. Each figure juxtaposes the empirical motif distribution with its corresponding power-law model, providing a straightforward visual comparison between TM and non-TM distributions at each threshold. In both TM and non-TM motifs, heavy tails are evident across all thresholds, indicating highly skewed frequency distributions where a few motifs are very prevalent, while the majority are rare. This trend matches the heavy-tailed patterns observed in the Zipf rank-frequency plots, reaffirming the consistency of this motif distribution pattern in both TM and non-TM data.

Despite this similarity, significant differences between TM and non-TM motif distributions become apparent from the power-law fits. Non-TM motifs follow a power-law distribution well across all thresholds, with stable exponents (α ≈ 3.76–3.79) and uniformly low KS distances (0.013–0.016). Likelihood ratio tests consistently showed no preference for the lognormal model (R ≈ 0.06, *p* > 0.9), confirming that non-TM motifs robustly follow a scale-free distribution characterized by a few dominant motifs and a long tail of rare ones.

By contrast, TM motifs show weaker adherence to a power-law. Their exponents are lower (~3.06 at TM ≥ 20%) and rise modestly at higher thresholds (3.10, 3.12, 3.18, and 3.16 for TM ≥ 40%, 60%, 80%, and 100%, respectively). KS distances are larger (0.032–0.038), and likelihood ratio tests consistently favored the lognormal distribution, with negative R values and significant or nearly significant *p*-values (e.g., R = −4.49 at TM ≥ 20%, *p* = 0.033; R = −2.52 at TM ≥ 100%, *p* = 0.075). Importantly, the closest convergence between power-law and lognormal fits occurs at TM ≥ 60%, where the discrepancy is smallest (R = −3.13, *p* = 0.059), suggesting partial alignment with scale-free behavior at this threshold.

Together, the scaling exponents, KS distances, and likelihood ratio tests indicate that non-TM motifs consistently follow scale-free dynamics, while TM motifs are generally better captured by lognormal distributions, with improved but still weaker power-law fits emerging around the 60% threshold. This transition point aligns with our Zipf and entropy analyses, highlighting TM ≥ 60% as a critical coverage level where motif distributions become more homogeneous and diverge most strongly from non-TM counterparts.

### 2.6. Shared Motif Analysis

**Figure 7:**
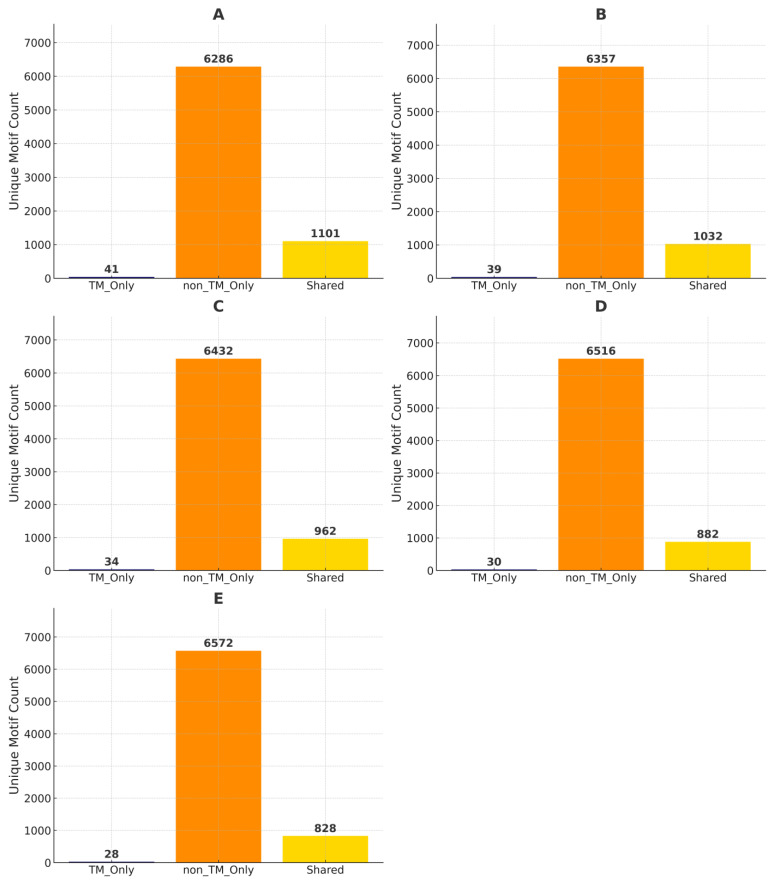
Unique GXXXG motif distribution for non-TM, shared, and TM. Each panel corresponds to a different TM percentage: (**A**) ≥20%, (**B**) ≥40%, (**C**) ≥60%, (**D**) ≥80%, and (**E**) 100%. The number of unique motifs is shown above each bar. Across all TM percentages, the non_TM_Only group exhibits the highest motif diversity, while TM_Only motifs are relatively scarce. The number of shared motifs gradually decreases with increasing TM percentage, suggesting that stricter TM constraints limit motif overlap between TM and non-TM proteins.

Figure 3’s analyses reveal that some GXXXG motifs are common to both TM and non-TM categories. To assess the uniqueness and overlap of these motifs, we compared the counts of motifs found exclusively in TM, exclusively in non-TM, or shared between both, across different TM coverage thresholds (20%, 40%, 60%, 80%, and 100%) as illustrated in Figure 7.

At TM coverage ≥ 20% (Figure 7A), the TM-only group contained only 41 unique GXXXG sequences, whereas the non-TM group exhibited 6286 unique motifs. Notably, 1101 motifs were found in both TM and non-TM sequences, suggesting a degree of conservation across membrane boundaries. As the TM coverage threshold increased, the number of TM-only motifs decreased steadily: 39 motifs at ≥40% (Figure 7B), 34 motifs at ≥60% (Figure 7C), 30 at ≥80% (Figure 7D), and just 28 at 100% TM coverage (Figure 7E). Conversely, non-TM unique motif counts increased slightly with higher TM stringency (ranging from 6286 to 6572), while shared motifs declined from 1101 to 828 as coverage thresholds increased.

This trend underscores that most GXXXG motifs are non-TM specific or shared with non-TM regions, with relatively few motifs exclusive to TM regions, even under strict TM coverage definitions. The steady decrease in shared motifs with increasing TM coverage also suggests that highly embedded TM motifs tend to diverge more from those found in non-TM regions. These findings highlight a constrained set of GXXXG motifs that are truly TM specific and suggest that shared motifs may function in both environments, while only a small subset is potentially specialized for stable integration within the lipid bilayer. Together, these results demonstrate the dominance of non-TM motifs in GXXXG diversity and emphasize the functional relevance of shared motifs across structural environments.

### 2.7. Zipf and Power-Law Analyses for Shared GXXXG Motif

**Figure 8:**
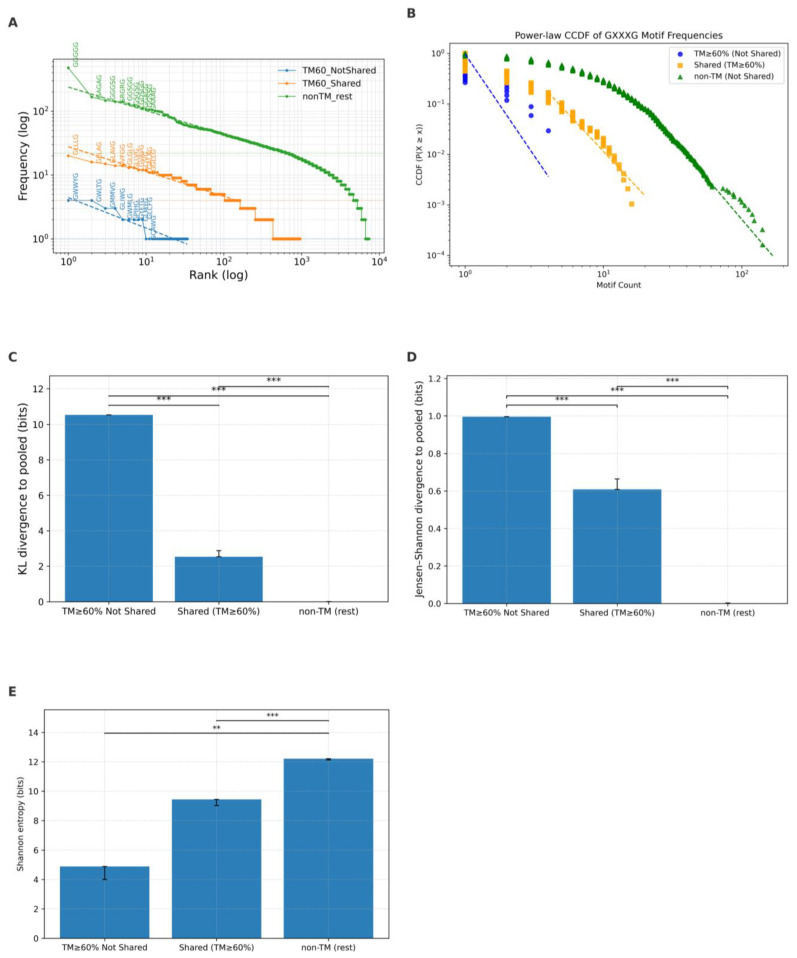
Statistical features of GXXXG motifs of TM-only, shared, and non-TM. (**A**) Zipf’s rank–frequency plot for TM-only, non-TM, and shared motifs at TM ≥ 60% with fitting curves (dashed lines). (**B**) Power-law CCDFs of motif counts across the three groups with fitting curves (dashed lines). (**C**) Kullback–Leibler divergence measurement. (**D**) Jensen–Shannon divergence calculation. (**E**) Shannon entropy values. Shared motifs show properties that are intermediate between TM-only and non-TM motifs. (** *p* < 0.01, *** *p* < 0.001).

To explore the sequence abundance characteristics of GXXXG motifs for the shared category, we conducted a comprehensive trend, Zipfian, and power-law distribution analyses, taking TM coverage ≥ 60% as a threshold. The results are visualized in Figure 8A–E for Zipf, power-law, KL divergence, JSD divergence, and entropy, respectively.

The rank–frequency distributions of GXXXG motifs across the three categories reveal distinct slopes in their Zipfian trends (Figure 8A). The estimated Zipf exponents were s ≈ 0.48 for TM ≥ 60% (blue curve) motifs, s ≈ 0.39 for shared motifs (orange curve), and s ≈ 0.37 for non-TM motifs (green curve). These differences indicate that TM ≥ 60% motifs exhibit the strongest skew, with a small number of highly dominant motifs, whereas non-TM motifs show the flattest slope, reflecting a more even distribution and greater sequence diversity. Shared motifs consistently fell between these extremes, suggesting partial constraint relative to TM motifs while retaining more diversity than TM-only regions.

The power-law modeling of motif count distributions further highlighted differences in tail behavior (Figure 8B). The maximum-likelihood estimated scaling exponents were α ≈ 4.71 for TM ≥ 60% (blue curve) motifs, α ≈ 3.92 for shared motifs (green curve), and α ≈ 4.22 for non-TM motifs (black curve). Notably, the non-TM category demonstrated the smallest KS distance (≈0.022) among the three groups (KS ≈ 0.11 and 0.20 for shared and TM, respectively), indicating that its distribution conforms most closely to a power-law model for non-TM, while TM and shared motifs showed the greatest deviations due to their broader diversity. Importantly, the unusually large α value for TM motifs is likely an artifact of the limited tail sample size (*n* < 50), which reduces statistical power and results in a poor fit compared to the shared and non-TM categories.

To quantify how much each motif distribution deviates from a uniform baseline, we computed KL divergence and JSD for each category (Figure 8C,D). KL divergence was highest for non-TM motifs at 0.44, reflecting their broad and unstructured diversity relative to a uniform reference. TM-only motifs had a lower KL divergence of 0.38, while shared motifs exhibited the smallest KL divergence at 0.35. Similarly, JSD was greatest for non-TM motifs (0.10), lower for TM-only motifs (0.082), and lowest for shared motifs (0.075). These results demonstrate that TM and shared motifs are more constrained and deviate less from an expected structured distribution than the more variable non-TM motifs.

Entropy, reflecting the overall diversity of sequences within each category, was highest in non-TM motifs, with an entropy value of approximately 12.2 bits (Figure 8E). TM-only motifs had much lower entropy at about 9.5, while shared motifs had an entropy similar to TM-only motifs at 9.7. These findings reinforce the notion that TM and shared motifs experience stronger constraints limiting their diversity, while non-TM motifs tolerate a wider range of sequences.

Together, the shared GXXXG motifs, observed in both TM (≥60%) and non-TM regions, exhibit intermediate statistical properties between TM-only and non-TM-only categories. Their Zipf exponent (s ≈ 1.10) and power-law α (≈1.78) indicate moderate skewness, suggesting partial constraint. Shared motifs also show lower KL divergence (0.35), JSD (0.075), and entropy (≈9.7) compared to non-TM motifs, reflecting reduced diversity. These findings imply that TM compatibility imposes selective pressure even on motifs present in both environments. The shared motifs likely represent a subset of sequences optimized to function under both structural contexts while retaining some sequence flexibility seen in non-TM regions.

### 2.8. The Analyses of Amino Acids Composition for GX1X2X3G Motif

**Figure 9:**
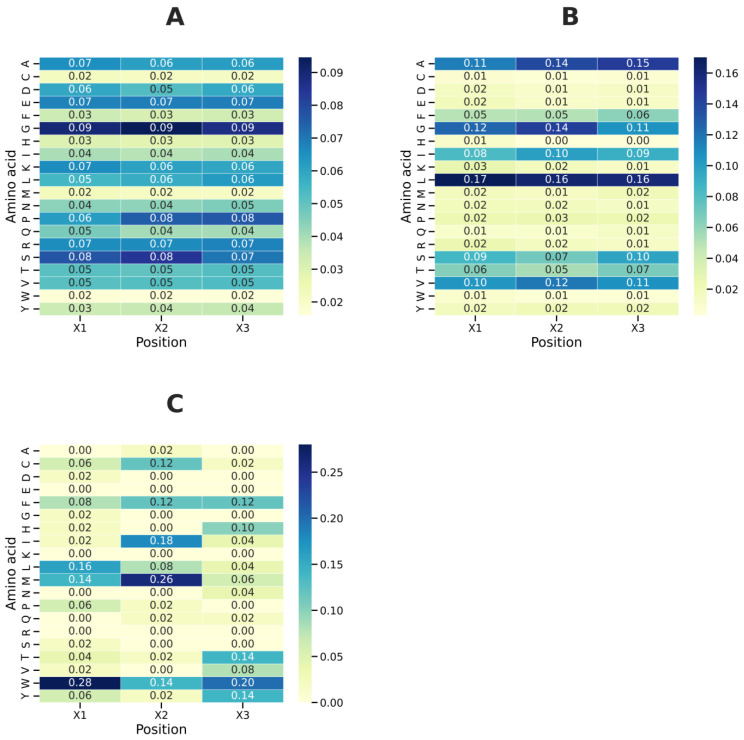
Amino acid composition of the X_1_, X_2_, and X_3_ positions in the GXXXG motif. Heatmaps illustrating log_2_ enrichment of amino acids: non-TM (**A**), shared (**B**), and shared TM (**C**). Hydrophobic and bulky residues (W, M, L, and I) are enriched in TM motifs, whereas flexible/polar/charged residues (G, S, E, and K) are predominant in non-TM motifs. Shared motifs show an intermediate amino acid composition.

To characterize the amino acid preferences around the GXXXG motif, we analyzed the distribution of residues at X1, X2, and X3 positions across three functional groups: non-TM, shared, and TM. The percentages were calculated from normalized frequency distributions, shown in Figure 9A–C, with each X-position scaled independently to total 100%.

For non-TM, the amino acid distribution in non-TM motifs was quite diverse, with significant presence of both small residues and charged side chains (see Figure 9A). At position X1, the most common residues were G (9%), followed by A, E, K, and R (7% each) and D and P (6% each). At position X2, G contributed roughly 9%, with S (8%), E and R (7% each), and A, K, and L (6% each). This varied profile indicates that non-TM motifs can include both polar and charged amino acids, aligning with their structural flexibility in water. At position X3, G (9%) was again most prevalent, with P (8%) next, followed by S, T, and E (7% each) and L, K, D, and A (6% each). The high levels of glycine and proline suggest a preference for flexibility, while the presence of charged residues like K and E shows they are suited for solvent-exposed structures.

Shared motifs show intermediate distributions between non-TM and TM groups, maintaining both small residues and hydrophobic amino acids (see Figure 9A). At position X1, leucine (L) is most prevalent (16%), followed by glycine (G) at 12%, alanine (A) at 11%, valine (V) at 10%, serine (S) at 9%, isoleucine (I) at 8%, and threonine (T) at 6%. This mix reflects contributions from both soluble-like small residues and hydrophobic side chains. At position X2, L is again dominant (16%), with G, A, V, phenylalanine (F), and S contributing 14%, 14%, 12%, 10%, and 7%, respectively. The prominence of L, G, A, and V indicates a tolerance for large and small side chains at this position. At position X3, leucine (16%) and alanine (15%) are most enriched, but G (11%) and S (10%) also play significant roles, along with V (11%). Thus, shared motifs do not exclusively prefer branched-chain hydrophobics; instead, they have a balanced composition of small residues (A, G, and S) and hydrophobic residues (L, V, and I). This distribution implies dual adaptability, aligning with their presence in proteins that may switch between soluble and membrane environments.

TM motifs were characterized by highly selective profiles dominated by bulky hydrophobic and aromatic residues (Figure 9C). At X1, W was the most enriched residue (28%), followed by L (16%) and M (14%). The prevalence of tryptophan underscores its role at membrane interfaces. At X2, M (26%) was predominant, accompanied by I (18%), W (14%), F (12%), and L (12%). This distribution emphasizes the contribution of methionine and aromatic side chains to helix–helix packing. At X3, W (20%) was again dominant, followed by Y, T (124% each), F (12%), and H (10%). Glycine was absent at this position. The exclusion of small residues and enrichment of bulky aromatics is consistent with structural constraints imposed by transmembrane helices.

A statistical test (Fisher’s exact test with FDR adjustment) was applied to assess the significance of the observed amino acid preferences at each position between groups. Positions X2 and X3 in TM versus non-TM comparisons showed the most significant differences. TM motifs strongly prefer small hydrophobic A (*p* < 0.0001) residues, particularly at X2, reinforcing their role in membrane insertion and helix packing. In contrast, non-TM motifs show an enrichment of polar/charged or helix-breaking residues (E and K with *p* < 0.0001).

The trend of increasing hydrophobicity from non-TM to shared to TM is visually evident in the enrichment heatmaps. While non-TM motifs tolerate polar/charged and flexible residues (G, S, E, P, and K), TM motifs strongly favor bulky hydrophobic residues (W, M, Y, and I), consistent with the physicochemical demands of membrane integration. Shared motifs bridge this gap, combining traits of both contexts (L, V, A, G, and S).

The clear enrichment of hydrophobic amino acids (M, W, L, I, V, and H) at X2 and X3 positions in TM motifs aligns with their role in stabilizing helix–helix interactions within the hydrophobic membrane core. The depletion of polar and flexible residues (S, T, Q, and D) in TM motifs suggests that these positions contribute critically to membrane association and are selectively disfavored in TM regions. Conversely, the presence of small and polar/charged residues at X_1_ in non-TM and shared motifs indicates a preference for flexibility and solvent accessibility outside the membrane environment.

Shared motifs appear to represent a transitional state, retaining some hydrophobic character required for membrane embedding while maintaining residues that allow interaction with aqueous environments. This duality may underpin the functional versatility of shared motifs.

## 3. Discussion

This study offers one of the most detailed and systematic analyses of the GXXXG motif so far, clarifying how its distribution, variety, and amino acid preferences differ across various structural environments. By combining structural annotations from UniProt and PDB with thorough statistical modeling, we reveal how evolutionary and biophysical constraints influence the characteristics of this adaptable motif.

Our results show a distinct split between GXXXG motifs in TM and non-TM regions. The non-TM motifs make up most of the dataset and are highly diverse in sequence, frequently featuring glycine-rich combinations, like GGGGG and GGGSG. These motifs likely contribute to flexibility, disorder, or dynamic interactions, aligning with the permissive sequence constraints and functional diversity of soluble regions. The observed high entropy, shallow Zipf exponent, and low power-law exponent for non-TM motifs indicate this diverse and adaptable pattern [22,23,24,25].

In contrast, TM motifs show significantly less diversity and are enriched with hydrophobic residues, especially leucine, isoleucine, and valine at the X positions. Motifs like GLLLG and GALAG become more common in TM regions, reflecting the physicochemical needs of membrane-embedded helices [26,27], which require tight packing and hydrophobic compatibility with the lipid bilayer. TM motifs have steeper Zipf slopes, higher power-law exponents, and lower entropy, suggesting a more selective and limited sequence set. These observations align with the structural demands of stable helix–helix interactions in the membrane, favoring a narrow range of effective sequences [1,2,3,4,26,27].

Shared motifs found in both TM and non-TM regions form a distinct subgroup with intermediate features. These motifs combine the hydrophobic enrichment typical of TM motifs with the broader compositional flexibility of non-TM motifs. Their moderate entropy, divergence, and intermediate Zipf and power-law values suggest they may serve as an evolutionary middle ground, optimized for function in both structural environments. This dual functionality could allow shared motifs to engage in dynamic interactions at the membrane–cytosol interface or to adjust to different structural needs based on cellular context. Although their exact roles are still unclear, their significant presence in our dataset highlights their potential biological importance.

A key outcome of this research is recognizing 60% TM coverage as a significant threshold for identifying TM-specific motifs that are biologically and statistically meaningful. At this level, TM motifs differ most from non-TM motifs, showing the lowest entropy and distinct statistical patterns, indicating that membrane constraints apply the strongest selective pressure beyond this point and push the repertoire toward highly specialized sequences. Lower thresholds include many motifs also found in soluble regions, reducing specificity, while higher thresholds unnecessarily limit the motif set without significant improvements in discrimination. Statistical analysis of motif frequency distributions supports this: TM motifs have heavy-tailed, skewed distributions consistent with strong selective constraints and limited diversity, while non-TM motifs have broader, more uniform distributions, suggesting a more permissive sequence space, with shared motifs in between. Power-law and Zipf analyses agree that TM ≥ 60% is the inflection point where TM motifs are most distinct from non-TM motifs, confirming its value as a reliable criterion for large-scale identification of TM-specific motifs.

Our analysis of amino acid positions reveals how structural context shapes motif sequences. TM motifs mainly prefer bulky hydrophobic residues like leucine, isoleucine, and valine at the X2 and X3 positions, emphasizing their importance in stabilizing membrane helices [1,2,3,4,26,27]. In contrast, non-TM motifs are richer in polar and flexible residues, such as serine and threonine, fitting their roles in soluble or disordered regions [28,29]. Shared motifs display intermediate patterns, blending hydrophilic and some hydrophobic traits that support partial TM compatibility. These significant patterns highlight the vital role of the X2 and X3 positions in defining structural and functional specificity. These position-specific preferences can serve as design constraints in protein engineering pipelines. For instance, the enrichment of leucine, isoleucine, or valine at X2/X3 in TM motifs provides a guide for engineering transmembrane helices with predictable oligomerization behavior. Conversely, motifs with glycine-rich or polar residues in non-TM contexts may be tailored for flexible or disordered protein segments.

The structural environment of GXXXG motifs is highly context-dependent: motifs in transmembrane proteins are predominantly found in helices, while those in soluble proteins occur more broadly in loops and coils. This structural dichotomy highlights the importance of analyzing membrane-associated and soluble motifs separately to avoid conflating distinct functional mechanisms.

From a biological perspective, these findings enhance our understanding of the structural and functional versatility of the GXXXG motif family. TM motifs are highly optimized for helix–helix association and structural integrity within the membrane, while non-TM motifs contribute to flexibility, conformational dynamics, and interactions in soluble regions [1,2,3,4,30,31,32,33]. Shared motifs likely play roles in processes that require adaptability, such as signaling, trafficking, or transient membrane association, where functional plasticity is advantageous.

These findings also prompt interesting questions regarding the role of shared motifs. Their intermediate features and common presence imply they could serve as versatile sequence elements, facilitating interactions in both membrane-bound and soluble environments. Additional research is needed to understand their precise functions, especially in dynamic or regulated activities at membrane–cytoplasm boundaries.

While this study relies solely on computational and statistical methods, it provides a strong foundation for future experimental validation and use. Techniques like site-directed mutagenesis or biophysical analysis of candidate motifs will be crucial to verify the biological importance of the identified TM-specific and shared motifs; however, this work does not include such validation. Still, the trends and benchmarks outlined here serve as a helpful guide for designing future experiments. Beyond fundamental research, our findings have direct implications for protein engineering and synthetic biology. The statistical benchmarks and sequence constraints revealed in this study offer a quantitative framework for rational motif design. Specifically, unique motifs enriched in TM versus non-TM settings can serve as building blocks for engineering helix–helix interfaces or disordered linkers.

Additionally, the motif-specific sequence statistics—such as position-wise amino acid biases—can inform feature extraction for machine learning models, including Hidden Markov Models (HMMs) for motif detection or diffusion-based generative models for de novo protein sequence design. These models could incorporate TM-coverage thresholds or motif entropy profiles to favor biologically relevant sequences. For example, motifs identified as “TM-specific” with low entropy and steep Zipf exponents could serve as training seeds in structure-conditioned generative networks, while shared motifs with intermediate divergence might be tuned for adaptive membrane–soluble interface applications.

This study has some limitations. It depends on existing database annotations, which might contain errors or inconsistencies, especially in TM region predictions. Our definition of TM coverage thresholds is operational and might not fully encompass all biologically relevant states. Although statistical patterns are evident, they show correlations, not causation; experimental validation is necessary to confirm functional insights. While experimental validation would reinforce these findings, this extensive computational analysis offers a valuable basis for future structural and functional research on GXXXG motifs.

Future research should broaden the analysis to include features like secondary structure predictions, solvent accessibility, and evolutionary conservation. Molecular dynamics simulations could provide deeper insight into how shared motifs behave and remain stable in various structural environments. Examining differences across taxa and protein families might uncover lineage- or function-specific patterns in motif utilization.

In conclusion, this study offers a thorough, quantitative overview of the GXXXG motif distribution across TM, non-TM, and shared environments. By defining TM ≥ 60% as a reliable cutoff for TM-specific motifs and exploring the unique features of shared motifs, we enhance our understanding of how sequence–structure relationships influence protein function. These findings underscore the glycine zipper as an evolutionarily flexible and structurally adaptable motif, connecting soluble and membrane environments and providing valuable opportunities for fundamental biological research and protein engineering.

## 4. Materials and Methods

### 4.1. Data Retrieval and GXXXG Motif Identification

We began this study by searching the UniProt Knowledgebase for protein sequences that feature the GXXXG motif. Using the UniProt REST API, we queried sequences from 10 to 25,000 amino acids, retrieving data in batches of 500 to manage server load and ensure completeness. A regular expression was employed to identify sequences containing the canonical GXXXG pattern. These sequences were then matched to structural data by cross-referencing UniProt IDs with PDB identifiers through the PDBe API (version 2.8.5). To ensure consistency, only the first PDB mapping for each UniProt ID was used, along with relevant chain information. The sequences and related metadata were stored in a pandas DataFrame and saved as a CSV file for further analysis. Additionally, each sequence’s UniProt XML file was parsed to gather detailed annotations, such as taxonomy and transmembrane (TM) regions.

### 4.2. Taxonomy Annotation and Redundancy Removal

To add biological context, each sequence was annotated with its full taxonomic lineage from UniProt XML data. We then applied a redundancy reduction pipeline. Sequences from the same organism that were identical, highly similar (≥95% identity), or linked to the same UniProt ID were marked as redundant. From each group of redundant entries, one representative was kept. This process produced two datasets: a non-redundant set with unique sequences and a redundant set with all entries clearly marked for redundancy. The pipeline was automated using Python, pandas, and Biopython.

### 4.3. Multiple Sequence Alignment and Mutation Variant Filtering

To exclude minor variants, we used Clustal Omega (version 1.2.4) [30] to perform multiple sequence alignments (MSAs) within each organism or UniProt group. We reviewed the alignments and filtered out sequences with ≥95% identity, keeping only representative and sufficiently diverse sequences. The alignment results were visualized to verify the effectiveness of the filtering.

### 4.4. Motif Extraction and TM Annotation

From the curated dataset, each GXXXG motif was accurately identified within its parent sequence, recording its position index. To determine each motif’s TM context, residue-level TM annotations from UniProt XML files were used. Each motif was given a TM coverage percentage based on the proportion of its residues that overlapped with annotated TM regions. Motifs were classified as TM if their coverage reached or surpassed certain thresholds (such as ≥20%, ≥40%, ≥60%, ≥80%, or ≥100%); otherwise, they were categorized as non-TM. Those found in both TM and non-TM contexts were labeled as “shared.” To ensure the reliability of TM classifications, we further evaluated UniProt’s TM annotations by distinguishing between “experimental” and “predicted” status using UniProt XML feature tags. For entries marked as “predicted,” we cross-referenced structural evidence from the Protein Data Bank (PDB) and AlphaFold2 models. TM regions lacking structural support in either source were excluded from the final dataset.

### 4.5. Frequency Analysis and Distribution Fitting

The counts of each unique GXXXG motif were calculated separately for TM-only, non-TM-only, and shared categories. Motif frequency distributions were visualized using bar plots and rank-frequency curves. To analyze their distributional features, we fitted the frequency data to Zipf-law and power-law models [22,23]. For Zipf’s law, rank-frequency data were log-transformed, and the exponent, *s*, was estimated through linear regression. Power-law fitting was performed using maximum-likelihood estimation with the powerlaw package, and Kolmogorov–Smirnov (KL) distances were calculated to evaluate the fit [31]. Additionally, likelihood ratio tests compared power-law and log-normal fits, with significance determined by *p*-values.

### 4.6. TM Coverage Threshold Analysis

To evaluate the impact of TM coverage definitions on motif classification, we systematically tested various thresholds (≥20%, ≥40%, ≥60%, ≥80%, and ≥100%). For each threshold, we calculated three metrics: TM ratio (TM motifs/total), non-TM ratio (non-TM motifs/total), and the TM/non-TM ratio. Line plots displayed how these metrics changed with increasing TM coverage cut-offs. We compared adjacent coverage levels using Chi-square tests of independence. The contingency tables for these tests included counts of TM and non-TM motifs, with *p* < 0.05 indicating significance. Results were directly annotated on the plots to emphasize key changes.

### 4.7. Statistical Divergence and Entropy Analyses

We used Shannon entropy to assess sequence diversity in each category. To compare motif distributions at various TM coverage thresholds against a non-TM baseline, we calculated information–theoretic divergence measures, including Kullback–Leibler (KL) and Jensen–Shannon divergence (JSD) [32,33]. KL divergence was measured in both directions (TM/non-TM and non-TM/TM) to account for asymmetries, while JSD served as a symmetric, bounded metric of dissimilarity. These metrics were displayed using heatmaps and line plots, with numerical values annotated.

### 4.8. Amino Acid Composition Analysis

The GXXXG motif was broken down into its individual positions: X1, X2, and X3. Amino acid frequencies at each of these positions were determined for TM-only, non-TM-only, and shared motifs. Heatmaps displayed the distribution of the 20 amino acids at each position across the three groups. To assess positional enrichment, we computed log_2_ enrichment scores for comparisons between TM and non-TM, shared versus TM, and shared versus non-TM. Positive values indicated over-representation, while negative values indicated depletion relative to the reference. Fisher’s exact test was used to evaluate the significance of differences in composition, with significant results (*p* < 0.05, *p* < 0.01, and *p* < 0.001) marked on the heatmaps.

### 4.9. Visualization and Output

All visualizations were created in Python (version 3.12) using matplotlib and seaborn. They include bar plots of motif counts, Zipf plots, power-law fits (using complementary cumulative distribution functions), entropy and divergence graphs, and heatmaps showing amino acid enrichment. Where relevant, statistical annotations such as *p*-values and trend lines were added. Top motifs were labelled directly on rank-frequency plots to emphasize essential sequences. The final figures were exported as high-resolution PNG images, and the results were summarized in CSV files to ensure reproducibility.

### 4.10. Software and Environment

All analyses were performed in Python (version 3.12) using pandas, numpy, scipy, Biopython, matplotlib, seaborn, powerlaw, and scikit-learn. Scripts ran in a standard desktop setup, with random seeds set where needed for reproducibility. All code and data pipelines are available upon request to promote transparency and reproducibility.

## Data Availability

The data are made available from the corresponding author upon request.

## Supplementary Materials

The following supporting information can be downloaded at: https://www.mdpi.com/article/10.3390/ijms26189014/s1.
