# Supplementary material for: Comprehensive Analysis of the GXXXG Motif Reveals Structural Context-Dependent Diversity and Composition Across Proteins
Source: Int J Mol Sci. 2025 Sep 16;26(18):9014. doi: 10.3390/ijms26189014 (PMC12470825; doi:10.3390/ijms26189014)
Supplement: Supplementary file 1 [file ijms-26-09014-s001.zip › ijms-3813492-supplementary.pdf]

Supplemental data

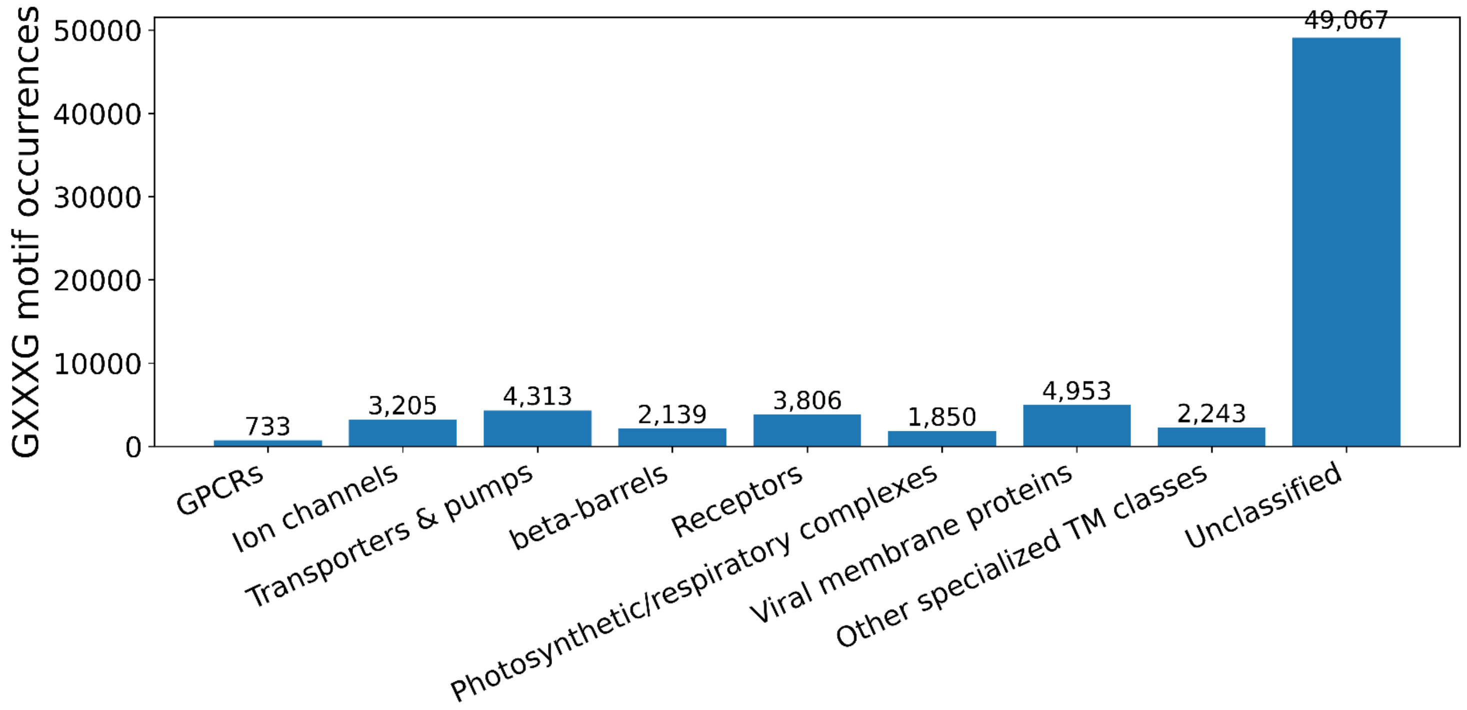

Figure S1. Distribution of GXXXG Motif Occurrences Across Transmembrane Protein Classes.

The bar plot shows the number of GXXXG motif occurrences classified into major transmembrane (TM) protein categories based on UniProt annotations. Categories include GPCRs, ion channels, transporters and pumps, β-barrels, receptors, photosynthetic/respiratory complexes, viral membrane proteins, and other specialized TM classes. A large portion (49,067 occurrences) falls under "Unclassified," indicating either non-TM or TM proteins without detailed subtype annotation. Motif counts are shown above each bar. This distribution highlights both functional diversity and annotation gaps in current TM protein databases.
